# Evolution of Eye Morphology and Rhodopsin Expression in the *Drosophila melanogaster* Species Subgroup

**DOI:** 10.1371/journal.pone.0037346

**Published:** 2012-05-25

**Authors:** Nico Posnien, Corinna Hopfen, Maarten Hilbrant, Margarita Ramos-Womack, Sophie Murat, Anna Schönauer, Samantha L. Herbert, Maria D. S. Nunes, Saad Arif, Casper J. Breuker, Christian Schlötterer, Philipp Mitteroecker, Alistair P. McGregor

**Affiliations:** 1 Department of Biological and Medical Sciences, Oxford Brookes University, Oxford, United Kingdom; 2 Institut für Populationsgenetik, Veterinärmedizinische Universität Wien, Vienna, Austria; 3 Howard Hughes Medical Institute, Princeton University, Princeton, New Jersey, United States of America; 4 Department of Ecology and Evolutionary Biology, Princeton University, Princeton, New Jersey, United States of America; 5 Champalimaud Neuroscience Programme, Champalimaud Centre for the Unknown, Lisbon, Portugal; 6 Department of Theoretical Biology, University of Vienna, Vienna, Austria; Vetmeduni Vienna Institute of Population Genetics, Austria

## Abstract

A striking diversity of compound eye size and shape has evolved among insects. The number of ommatidia and their size are major determinants of the visual sensitivity and acuity of the compound eye. Each ommatidium is composed of eight photoreceptor cells that facilitate the discrimination of different colours via the expression of various light sensitive Rhodopsin proteins. It follows that variation in eye size, shape, and opsin composition is likely to directly influence vision. We analyzed variation in these three traits in *D. melanogaster*, *D. simulans* and *D. mauritiana*. We show that *D. mauritiana* generally has larger eyes than its sibling species, which is due to a combination of larger ommatidia and more ommatidia. In addition, intra- and inter-specific differences in eye size among *D. simulans* and *D. melanogaster* strains are mainly caused by variation in ommatidia number. By applying a geometric morphometrics approach to assess whether the formation of larger eyes influences other parts of the head capsule, we found that an increase in eye size is associated with a reduction in the adjacent face cuticle. Our shape analysis also demonstrates that *D. mauritiana* eyes are specifically enlarged in the dorsal region. Intriguingly, this dorsal enlargement is associated with enhanced expression of *rhodopsin 3* in *D. mauritiana*. In summary, our data suggests that the morphology and functional properties of the compound eyes vary considerably within and among these closely related *Drosophila* species and may be part of coordinated morphological changes affecting the head capsule.

## Introduction

Compound eyes are composed of subunits called ommatidia and the great diversity of eye sizes and shapes among insects [1] is mainly the result of evolutionary changes in either ommatidia number (1 in some ant workers to 30000 in dragonflies) or ommatidia diameter (5–50 µm) [1]–[3]. These two parameters significantly influence the optical properties of the compound eye. The larger the ommatidia diameter the more light can be captured. However, the higher sensitivity of larger lenses has a negative impact on the resolution achieved, which is improved with decreasing diameter [4]–[8]. Therefore, a compound eye that is composed of few ommatidia with large lens diameters is highly sensitive to low light levels but the correspondingly large interommatidial angles result in low acuity. Conversely, the same size of eye made up of many smaller ommatidia would have reduced interommatidial angles and, therefore, could provide higher acuity. This trade-off between sensitivity and acuity is further complicated by the fact that the resolution is limited by diffraction when ommatidia get smaller [9]. Therefore, a vast number of specializations in insect compound eye composition have evolved in order to balance these constraints and optimize vision for specific needs. For example, dorsal-frontal locally restricted acute zones with large ommatidia and reduced inter-ommatidial angles that produce increased resolution to optimise the pursuit and capturing of prey or mating partners have evolved in several insects [9]–[13].

Acute zones with larger ommatidia, however, require more space within the eye, and indeed within the whole head capsule, resulting in the formation of diverse eye and head shapes. In males of the common house fly, *Musca domestica*, the frontal-dorsal acute zone in each eye, the so-called “love spots” [10], [14] are associated with dorsally enlarged eyes compared to females. A similar sexual dimorphism in size of the dorsal part of the eye is also observed in other dipterans [10], [15]. Interestingly, the frontal-dorsal enlargement of male eyes in these flies is associated with a reduction in the face cuticle tissue between the eyes suggesting that there may be a developmental trade-off between retinal and face cuticle tissue [15]. A negative correlation of retinal field and face size has been reported previously in both males and females of several *Drosophila* species, including *D. melanogaster* [16]–[18], and it is in this species that compound eye development and functional morphology is best understood [19].

In addition to optical properties like sensitivity and acuity, key experiments in *D. melanogaster* have shown that the ability of these flies to detect different fractions of the light is heterogeneously distributed throughout the eye [20]–[23]. Each ommatidial unit consists of a cluster of eight light sensitive photoreceptor (PR) cells (R1–R8) and surrounding associated cone and pigment cells that form the lens and isolate the PR cells from the light coming from neighbouring ommatidia [19], [24]. Depending on their morphology and functional properties, the PR cells can be subdivided into outer PRs (R1 to R6) and inner PRs (R7 and R8). The outer PRs are organized in a chiral trapezoid and in the center of this ring, the inner PRs are located on top of each other. The PR cells have extensively folded membranes, the rhabdomeres, containing various light sensitive Rhodopsin proteins, which prescribe the functional properties of the PRs [19]. All outer PRs express the broad range Rhodopsin 1 (Rh1) that enables motion detection and facilitates vision in dim light [24], [25]. The inner PRs enable colour vision and the detection of polarized light. The expression of different Rhodopsins in these cells determines the various ommatidia subtypes. Approximately 30% of all ommatidia are of the “pale” type and express the UV-sensitive Rhodopsin 3 (Rh3) in R7 and the blue-sensitive Rhodopsin 5 (Rh5) in R8 enabling them to discriminate among short wavelengths. The remaining 70% “yellow” type ommatidia detect longer wavelengths by expressing the UV-sensitive Rhodopsin 4 (Rh4) in R7 and the green-sensitive Rhodopsin 6 (Rh6) in R8. Both ommatidia types are distributed stochastically in a ‘salt-and-pepper’ pattern [23], [26]–[32].

In the dorsal portion of the eye further ommatidia subtypes can be found including a subset of the “yellow” type ommatidia (10% of all ommatidia), which co-express both UV-sensitive Rhodopsins Rh3 and Rh4 in R7 [33]. Further UV sensitivity is achieved in the ommatidia of the dorsal rim area (DRA) where R7 and R8 both express Rh3 [29], [34].

While several previous studies have reported variation in ommatidia number in species of the *D. melanogaster* complex [35], [36] that may be indicative of functional differences in vision, the extent of intra-specific and inter-specific variation in important traits such as eye morphology and Rhodopsin expression in these species is not well understood. A more detailed understanding of the patterns of variation in eye morphology is likely to inform and encourage studies of the evolutionary processes that produced them. Therefore we have characterised variation in eye size and shape, and the corresponding expression of *rhodopsins* in different strains of *D. melanogaster*, *D. simulans* and *D. mauritiana*.

We find that *D. mauritiana* generally has larger eyes than its sibling species resulting from either larger ommatidia or more ommatidia in different strains of this species. We also found extensive differences in eye size among *D. melanogaster* and *D. simulans* strains caused mainly by variation in ommatidia number. In all three species, we observed that enhanced eye size is generally established at the expense of adjacent face tissue. Furthermore, the eyes of *D. mauritiana* are also dorsally enlarged relative to the other species and this is associated with enrichment in the expression of *rhodopsin 3*, which is predominantly expressed in dorsal specific ommatidia types. Taken together our results evidence that there is extensive variation in eye morphology and *rhodopsin* expression in *Drosophila* that could cause differences in vision within and among these species.

## Materials and Methods

### Fly culture, dissections and microscopy

The following *Drosophila* species and strains were used for the experiments: *D. melanogaster* (M36 (14021-0231.36, genome stock), Oregon R (OreR), Zi372 (a gift from John Pool)), *D. simulans* (*y, v, f* (YVF), *w*501, Kib32) and *D. mauritiana* (TAM16, MAV1, *w^−^*). All flies were raised on the same standard cornmeal diet at 25°C with a 12 hours dark/light cycle. Flies for the *rhodopsin* qPCR experiments were raised in complete darkness. The density was controlled during larval development by limiting the content of each vial to 40 to 50 freshly hatched L1 larvae. The resulting adult flies of each strain were then pooled from multiple vials, and analyzed 3 to 7 days after eclosion.

Additional *D. simulans* and *D. mauritiana* strains that were raised at room temperature, but not controlled for larval density were also surveyed. Information on these strains is available on request.

Flies were decapitated and the heads mounted on sticky tape facing upwards. A first leg was also removed from all specimens, and additionally a wing was taken from *D. simulans* YVF, *D. mauritiana* TAM16 and *D. melanogaster* M36 strains (note that left or right wings and legs were taken randomly). Legs and wings were mounted in Hoyer's medium. All images were recorded using a Leica DM5500 compound microscope or a Leica M205 stereo microscope and a DFC300 Camera.

For SEM images, flies were collected in 70% ethanol. After cutting specimens midway through the thorax, their heads were dehydrated in a graded ethanol series and critical point dried in CO_2_ (Tousimis Samdri-780). The dried samples were mounted on stubs with one eye oriented upwards next to the corresponding first leg. Stubs were sputter-coated with gold (Polaron coater) and SEM images were taken on a Hitachi S3400N.

### Measurements

All statistical analyses and graphical outputs for the measurements described below were performed and generated using R [37].

### Head and eye measurements

**Figure 1:**
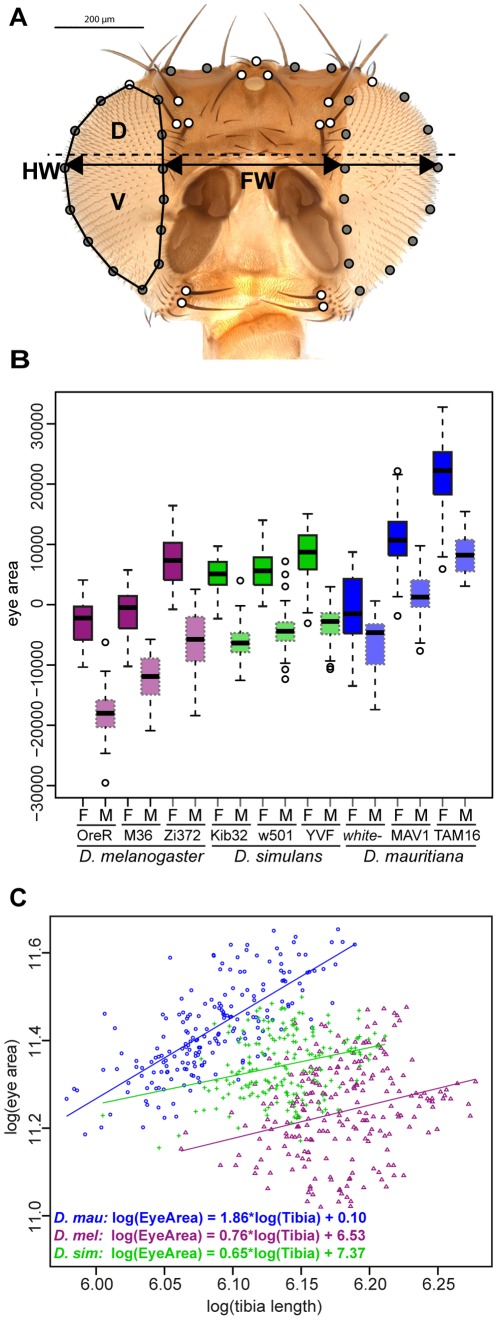
Eye size variation in different strains of three *Drosophila* species. **A.** Frontal view of a *D. melanogaster* head. The fifteen white filled circles represent anatomical landmarks (bristle insertions, intersections of the outlines of the eyes with the dorsal margin of the head capsule, dorsal most part of the head capsule between the paired ocelli) and the grey filled circles represent semi-landmarks on curves (eye outlines, dorsal outline of the head). The eye area was defined as the area of the polygon defined by the landmarks around the eye (see left eye). The dotted line illustrates where the eyes were separated into a dorsal (D) and ventral (V) portion for manual measurements of dorsal and ventral eye area. The black arrows represent linear measurements taken to assess face width (FW) and head width (HW). **B.** Eye area variation in three *Drosophila* species. Eye area is given as residuals of a regression of eye area in µm^2^ and tibia length to account for variation in body size. Females (F) and males (M) are shown separately for each strain. See also Table S3 for the results of pair-wise comparisons. **C.** Scaling relationships for the three species analyzed. Females and males of all three strains per species were pooled and log-transformed eye areas were plotted against log-transformed tibia length. Linear regressions are shown for each species and the respective regression equation is given. The slope of each regression corresponds to the allometric coefficient α.

Head and eye measurements were obtained using two approaches. First, for *D. simulans* YVF, *D. melanogaster* M36 and *D. mauritiana* TAM16, we manually measured the whole eye area, dorsal (D) and ventral (V) eye area, and the face width (FW) with the *Analysis* tools of Adobe Photoshop CS5 (Figure 1A). For dorsal and ventral eye area measurements, the eye was divided along the dotted line shown in Figure 1A, which intersects the cuticular bulge dorsal of the antenna. Differences in the ratio of dorsal to ventral regions of the eye between species were tested using a Kruskal-Wallis rank test followed by pairwise Wilcoxon rank tests. Eye area and face size were also extracted from landmark annotated frontal head pictures of all specimens analyzed. In this case, eye area was defined as the area enclosed by the polygon that arises by connecting the landmarks and semi-landmarks around the eye (see Figure 1A, left eye) or face area. Since whole eye areas measured manually or extracted from landmarks were highly correlated (Spearman's rank correlation: *r* ^2^ = 0.98, *p*<2.2×10^−16^), we only report the landmark based eye areas for all strains here. Eye size is reported as residuals of eye area and tibia length (to control for body size variation between individuals, see below). Differences in eye size between strains were tested using ANOVA followed by Tukey comparisons.

For *D. simulans* and *D. mauritiana* strains examined in our broader survey of additional strains, eye size in five males and five females for each strain was measured as eye width (see Figure 1A; head width HW – face width FW = eye width). Note that HW and FW are defined by the intersection with the dorsal most part of the antennae (Figure 1A).

### Tibia and wing measurements

In order to estimate the body size of the individuals we also measured the length of tibia of the first leg. In addition, we analyzed three traits in one wing of each individual of *D. melanogaster* M36, *D. simulans* YVF and *D. mauritiana* TAM16 flies. Fifteen landmarks, which were placed on vein intersections and around the wing margin (see Figure S1), were recorded using ImageJ and a custom plugin [38]. Wing size was then estimated by calculating the centroid size based on raw landmark coordinates for each wing using MorphoJ [39]. Since the use of either wing size or tibia length to account for body size (e.g. residuals of regression lines between body size and eye area) resulted in the same eye size pattern between strains and species, we used tibia length as a proxy for body size consistent with previous studies [40], [41] with the exception of the strains examined separately in our broader survey (Figure S2), where the residuals of regression lines between eye width and the distance between the wing landmarks 9 and 13 were calculated (Figure S1).

### Ommatidia count and facet surface area measurements

**Figure 2:**
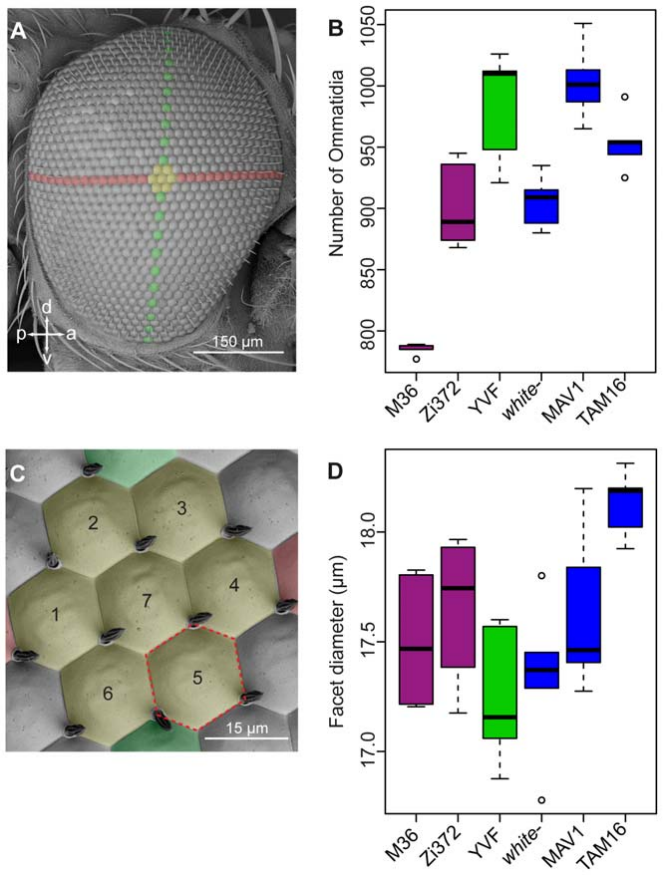
Ommatidia number and ommatidia diameter in three *Drosophila* species. **A.** SEM micrograph of the right eye of a female *D. simulans* fly. The central a-p row is marked in red, the central d-v row is marked in green and the central ommatidia are marked in yellow. Anterior is to the right. **B.** Total ommatidia number per eye. Box plots illustrate the distribution of ommatidia numbers of five females per strain. **C.** A rosette of seven central ommatidia. Colour markings correspond to those in A. The red-dotted hexagon around ommatidium 5 is shown as an example of how the surface area of a single ommatidium was determined. **D.** Central ommatidia diameter (mean of seven central facets per specimen) based on ommatidium surface area. Box plots depict five females per strain.

The total number of ommatidia per eye was counted manually from SEM images of the entire eye from each specimen. Using these images the centre of the eye was defined by first counting the number of antero-posterior (a-p) rows of ommatidia and by then intersecting the centre a-p row (marked red in Figure 2A) with the centre dorsal-ventral row (marked green in Figure 2A), which starts and ends in the approximate centre of the a-p rows at the dorsal and ventral extremes of the eye. The number of a-p and dorso-ventral (d-v) rows are summarized in Table S2 for *D. melanogaster* (M36, Zi735), *D. simulans* (YVF) and *D. mauritiana* (TAM16). For high-magnification (1.5 to 1.6 k) SEM images of the centre of the eye, the specimen was carefully tilted to minimize perspective projection distortion. Images of the central ommatidia were taken in SE mode at a working distance of 11 to 13 mm. For each eye, the area of each ommatidium from a rosette of seven (marked yellow in Figure 2A,C) was measured using the ImageJ polygon selection tool, as illustrated in Figure 2C. The mean area of seven ommatidia per eye was transformed to mean ommatidia diameter via 
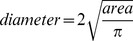
, thus approaching it as the area of a circle. Differences in ommatidia diameter between species were tested for using ANOVA. In addition, the ommatidia size was estimated for at least five males and five females of *D. melanogaster* (M36, Zi735), *D. simulans* (YVF) and *D. mauritiana* (TAM16) by dividing the total eye area (as measured on SEM pictures of lateral eye views) by the number of ommatidia for the same specimen (see Table S2). Note that this estimate of ommatidia size was consistent with the direct measurements of ommatidia size made in females as described above.

### Scaling relationships

To investigate if scaling relationships between eye size and tibia length change between species we regressed log-transformed eye area values over log-transformed tibia length measurements for each species (males and females of all three strains per species pooled). In the resulting linear equation 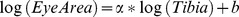, the slope α is the allometric coefficient [42]–[44]. We then tested for differences between species using an ANCOVA.

### Morphometrics

On frontal images of each head 45 landmarks were digitized using the software tpsDig2 [45] (Figure 1A). Fifteen landmarks are homologous anatomical landmarks (white dots in Figure 1A); both coordinates of these landmarks are determined by the specimens' anatomy. The remaining 30 landmarks are semi-landmarks on curves (outlines of the eyes and upper outline of the head; grey dots in Figure 1A). Semi-landmarks are constrained to lie on the corresponding curve, but the position along the curve cannot be located unambiguously. In an iterative algorithm they are thus allowed to slide along the curve until the bending energy between each individual and the sample mean form is a minimum [46], [47]. Bending energy is a measure of the total amount of local deformation that is necessary to transform one landmark configuration into another. Through the sliding process the semi-landmarks acquire geometrically homologous locations within the sample and can be treated in the same way as the anatomical landmarks in the subsequent statistical analysis.

All 1168 landmark configurations were superimposed by a Generalized Procrustes Analysis, standardising for position, orientation, and overall size of the configurations [48], [49]. The resulting Procrustes shape coordinates thus capture information on the shape of the landmark configurations only. Overall size of the landmark configurations is measured as Centroid Size (the square root of the summed squared distances from each landmark to the centroid).

The effects of two principal components (PCs) of the within-population distribution of the shape coordinates reflecting variation in the orientation of the head (not shown) were removed by projecting the data into the subspace perpendicular to these two principal components (this is equivalent to considering only the subsequent principal components, thus, thereafter here called PC 1 and PC 2). In addition, the resulting landmark configurations were symmetrised by averaging each configuration with its relabelled reflection [49]–[51].

We analysed the pattern of differences between sex-specific population mean shapes by a principal component analysis (PCA) of the adjusted shape coordinates. We visualized shape differences by thin-plate spline (TPS) deformation grids and quantified the amount of differences in terms of Procrustes Distance [52]. The statistical significance of group mean differences was estimated by permutation tests based on 10000 random permutations [53].

All morphometric and associated statistical analyses were carried out in Mathematica 8.0.

### Quantitative real-time PCR (qPCR)

For the qPCR we used females of *D. melanogaster* OreR, *D. simulans* Kib32 and *D. mauritiana* TAM16. Flies raised as described above were collected 3 to 5 days after eclosion and placed in 15 ml tubes at −80°C. Approximately 100 to 200 flies were decapitated by vigorously shaking the tubes and subsequently the heads were separated from the other body parts with no. 25 and no. 40 brass sieves [54]. We extracted RNA from those heads with standard kits (RNeasy, Qiagen) and the reverse transcription was carried out with the First Strand cDNA synthesis Kit and the Oligo(dT)_18_ Primer (Fermentas). The quantity and quality of the extracted RNA and the synthesized cDNA was controlled by concentration measurements using the fluorescent dye based Qubit technology (Invitrogen). cDNA synthesis and qPCR was performed with the same amount of RNA and cDNA, respectively, for each of the three species.

Primers for qPCR were designed to amplify short 60 to 75 bp fragments for each of the seven *rhodopsin* (*rh*) genes. Additionally, the primers were designed to span exon-exon boundaries to prevent the amplification of potential genomic DNA contamination (exception: *rh3* and *rh7*). For all *rh* genes except *rh6* the same assay was used for all three species. Due to a single nucleotide difference in the most conserved region of the three *rh6* sequences, three individual assays were used.

qPCR reactions based on the EvaGreen dye (Solis BioDyne) were performed twice for three independent cDNA samples for each opsin and species to account for variation in cDNA synthesis and well-to-well differences. Non-template controls (cDNA synthesis without RNA template) and RT- controls (cDNA synthesis reactions where the Reverse Transcriptase was replaced by water) were performed as negative controls. For each cDNA template and assay a standard curve with 3 serial dilutions covering a 64-fold range was defined to calculate template specific PCR efficiencies (E) [55]. We determined the critical cycle number (ct) for 10 to 12 replicates for each opsin and species from which the respective expression level was calculated: 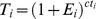. We first calculated relative expression levels for each *rhodopsin* gene as a fraction of the sum of total *rhodopsin* expression [55]–[58]:
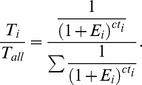
Since *rh1* is expressed in R1 to R6 of all ommatidia and expression level represents approximately 74% of all seven opsins in both *D. melanogaster* and *D. mauritiana*, we related the expression levels of *rh2-rh7* to *rh1* (T_i_/T_rh1_) for the analysis. Note that we were not able to detect the expression of *D. simulans* Kib32 *rh5* unambiguously, hence we omitted this opsin from the analysis.

The data from this study has been deposited in the Dryad Repository: http://dx.doi.org/10.5061/dryad.q8758

## Results

### Eye size variation in the *D. melanogaster* species subgroup

To survey eye size in *D. melanogaster*, *D. simulans* and *D. mauritiana*, we measured eye area in three strains of each of these three species (Figure 1A). Overall body size differences between strains and sexes were accounted for by normalizing eye measurements by the length of the tibia of the first leg.

We found that the *D. mauritiana* strains TAM16 and MAV1 have larger eyes than all *D. melanogaster* and *D. simulans* strains surveyed (Figure 1B, Table S1; one-way ANOVA: F(17,597) = 168.37, p<0.0001). A broader survey of eye width variation using additional *D. mauritiana* and *D. simulans* strains further indicates that *D. mauritiana* tends to have larger eyes (Figure S2). However, we did find an exception to this pattern because the white-eyed strain of *D. mauritiana* has significantly smaller eyes than the other strains of *D. mauritiana* and falls within the range of eye areas of the other two species (Figure 1B, Table S1).

We found that *D. melanogaster* M36 and OreR have the smallest eyes among the strains surveyed, however the African strain of this species (Zi372) exhibits eye areas comparable to those of the *D. simulans* strains (Figure 1B, Table S1; *D. melanogaster* females of M36 and OreR are not significantly different). In contrast to the variation observed among strains of *D. mauritiana* and *D. melanogaster*, the *D. simulans* strains Kib32, w501 and YVF did not differ significantly (Figure 1B, Table S1), although our broader survey of additional strains does suggest that there is also considerable eye size variation in this species (Figure S2).

Given the eye size differences we observed among *D. melanogaster*, *D. simulans* and *D. mauritiana*, we next investigated the scaling relationships between eye size and body size as represented by tibia length [42]–[44], [59]–[61]. We found that the larger eyes of *D. mauritiana* are associated with a positive allometry (hyperallometry) (allometric coefficient: α = 1.86, Fig 1C). In contrast, eye size exhibits negative allometry in *D. simulans* and *D. melanogaster* with allometric coefficients of α = 0.65 and 0.76, respectively (Figure 1C). While the main differences between the linear scaling relationships of *D. simulans* and *D. melanogaster* is their intercept (*D. simulans:* 7.37; *D. melanogaster:* 6.53), the linear correlation of eye area and body size in *D. mauritiana* is mainly characterized by a difference in the slope (allometric coefficient) compared to the other two species (Figure 1C). An ANCOVA for the whole dataset ((between-subject factor: species (*D. melanogaster, D. simulans, D. mauritiana*); covariate: body size (log(tibia))) reveals main effects of species (F(2,612) = 313.92; *p*<0.0001), body size (F(1,612) = 158.09; p<0.0001) and a significant interaction between body size and species (F(2,612) = 18.33; p<0.0001). ANCOVA analyses for species pairs shows that the slopes for *D. melanogaster* and *D. simulans* (interaction between species and body size: F(1,421) = 0.2; *p* = 0.66) are not significantly different, while the slopes between *D. melanogaster* and *D. mauritiana* (interaction between species and body size: F(1,422) = 24.00; *p*<0.0001) and *D. simulans* and *D. mauritiana* (interaction between species and body size: F(1,381) = 37.34; *p*<0.0001) are significantly different. Therefore, the larger eyes of *D. mauritiana* are associated with changes in the scaling relationships of body parts between this species and *D. simulans* and *D. melanogaster*.

Our analysis of eye size variation also revealed a consistent pattern of sexual dimorphism across all species with females having significantly larger eyes than males even after correcting for the larger body size of females (Figure 1B, Table S1).

### Variation in eye size is due to variation in ommatidia number and ommatidia size

Differences in the overall size of compound eyes can be caused by variation in the number and/or size of the ommatidia, and, therefore, we measured these parameters in females of several strains of *D. melanogaster*, *D. simulans* and *D. mauritiana* for which we surveyed eye area (Figure 2A,C).

*D. mauritiana* TAM16, the strain with the largest eye area in our survey, has on average 954 (±24) ommatidia in each eye (Figure 2B), which is surprisingly slightly fewer than both *D. mauritiana* MAV1 (1004±32) and *D. simulans* YVF (983±46), which have smaller eyes than those of *D. mauritiana* TAM16 (Figure 2B). However, central ommatidia size in *D. mauritiana* TAM16 is significantly larger than in *D. simulans* YVF (one-way ANOVA: F(5,23) = 4.66; p = 0.004) (Figure 2C,D), showing that the eye size differences between these two strains of *D. mauritiana* and *D. simulans* are mainly a consequence of ommatidia size differences. Ommatidia size between the two *D. mauritiana* strains, TAM16 and MAV1, is not significantly different, while TAM16 has significantly larger ommatidia than the *D. mauritiana w* ^−^ strain.

The differences in eye size between *D. melanogaster* and *D. simulans*, and among different strains of these two species, appears to be mostly due to ommatidia number (Figure 2B), rather than ommatidia size variation (Figure 2D). For example, ommatidia size is similar between *D. melanogaster* strains M36 and Zi372, but the latter has on average 117 more ommatidia in each eye and therefore larger eyes overall (Figure 1B, 2B,D).

Interestingly, differences in the total number of ommatidia between large and small eyes among all strains and species results from differences in the number of rows along both the a-p and d-v axes (Table S2).

To further explore whether the sexual dimorphism in eye size that we observed is caused by differences in either ommatidia number or size, we counted the number of ommatidia and estimated their size in both males and females of *D. melanogaster* (M36, Zi735), *D. simulans* (YVF) and *D. mauritiana* (TAM16) (Table S2). This data clearly illustrates that females possess more and larger ommatidia than males (Table S2), suggesting that differences in both traits underlie the observed sexual dimorphism in eye area.

### Analysis of relative head proportions using geometric morphometrics

**Figure 3:**
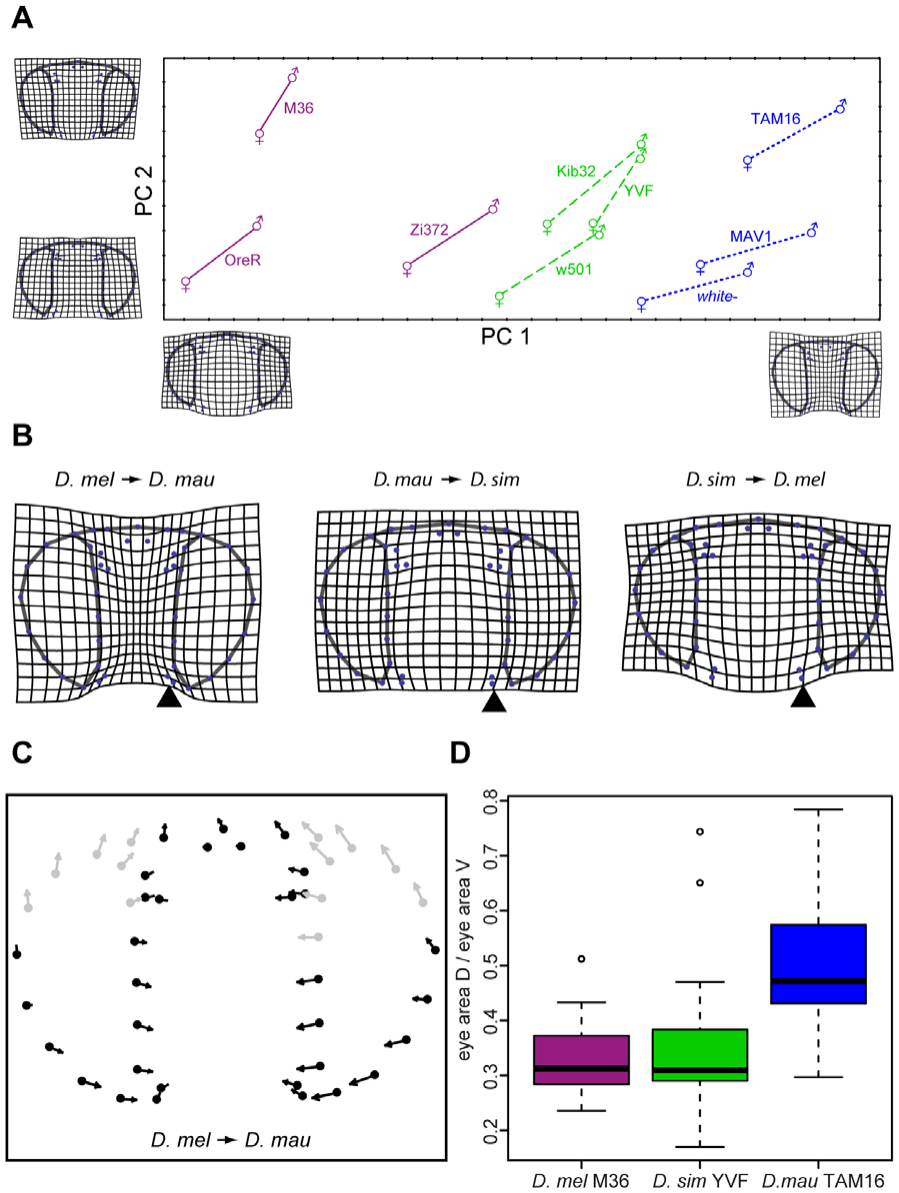
Eye and head shape differences between three *Drosophila* species. **A.** Principal component (PC) scores of the strain- and sex-specific average shapes after correcting for head posture. The male and female averages of each strain are connected by a line (*D. melanogaster* strains: solid red, *D. simulans* strains: dashed green line, *D. mauritiana* strains: dotted blue). The deformation grids represent the deformations from the mean shape to the positive and negative ends of the corresponding PC axis, amplified by a factor of 2. **B.** Deformation grids showing shape differences between the three species means, amplified by a factor of 2. Left panel: *D. mauritiana* compared to *D. melanogaster*. Middle panel: *D. simulans* compared to *D. mauritiana*. Right panel: *D. melanogaster* compared to *D. simulans*. Arrowheads indicate the position of the ventral bristles. **C.** Eye shape variation in the dorsal region of the retinal field of *D. mauritiana* compared to *D. melanogaster* depicted by vectors of relative landmark displacement. Relatively long vectors in the dorsal portion that point towards the median-dorsal region of the head are highlighted in red). **D.** Box plot of the ratio of dorsal eye area (D) and ventral eye area (V). The areas were measured manually in ten males and ten females of the three strains.

We next investigated whether differences in eye size among *D. melanogaster*, *D. simulans* and *D. mauritiana* were associated with other changes in the head of these flies resulting in differences in proportions of head structures or shape differences. To do this we used a geometric morphometrics approach based on 45 landmarks and semi-landmarks. After correcting for head posture, the first two principal components (PCs) of the strain- and sex-specific average shapes were visualized in a scatter plot (Figure 3A). PC 1 and PC 2 account for 89% of landmark variation between these average configurations.

The TPS deformation grids show that PC 1 reflects differences in the size of the eyes relative to the size of the face (eye-to-face) ratio and the different strains cluster according to their species along this PC (Figure 3A). To further quantify this pattern we extracted face and eye measurements for flies from all nine strains from the landmarks. Strains with small eye areas have the smallest eye-to-face ratio and vice versa (Table S3). For example, *D. melanogaster* OreR, which has the smallest eye area, exhibits the smallest eye-to-face ratio, while *D. mauritiana* TAM16, which has the largest eyes has the largest eye-to-face ratio (Table S3). Furthermore when we compared the eye-to-face ratio across the three species, *D. mauritiana* generally has the largest eyes relative to the face, although *D. mauritiana w^−^* is more clearly separated from *D. simulans* by PC 1 than by just eye-to-face ratio alone (Table S3). *D. melanogaster* strain Zi372 is clearly different from the other two *D. melanogaster* strains in terms of eye-to-face ratio (Figure 3A and Table S3) consistent with the larger eye area of this African strain compared to the two other strains of *D. melanogaster* used in our study (Figure 1B).

These descriptions of relative eye and face size from our morphometric analysis were corroborated by manual measurements of face width and eye area in females of one strain per species (*D. mauritiana*: TAM16, *D. melanogaster*: M36, *D. simulans*: YVF) (see black arrow in Figure 1A for FW measurement). The eye area divided by the squared value of the face width shows that *D. mauritiana* TAM16 has the largest eye-to-face ratio (0.74±0.035) while *D. melanogaster* M36 (0.45±0.032) and *D. simulans* YVF (0.57±0.049) possess much smaller ratios (all ratios are significantly different; one-way ANOVA: F(2,145) = 779.88, p<0.0001). Note that these observed differences in eye-to-face ratios are not only due to variation in eye size, since the face width is significantly reduced in *D. mauritiana* compared to *D. simulans* and *D. melanogaster* (Figure S3; ANOVA: F(2,145) = 80.33, *p*<0.0001).

PC 2 reflects differences along the d-v axis of the face. The main variation concerns the width of the dorsal and most prominently the ventral face region (see TPS deformation grids at the y-axis in Figure 3A). While neither species nor strains cluster along PC 2, this PC mainly explains shape differences between females and males found in all strains of all three species (PC 2 in Figure 3A). For these frontal images, males and females mainly differ in the relative size (eye-to-face ratio, see also Table S3), the shape, and the orientation of the eyes. The most obvious difference, however, is evident along the d-v axis in the face region. While females have wide ventral face regions, male ventral faces are much narrower as shown by more laterally located ventral bristles in females (PC 2 in Figure 3A). A permutation test confirms that male and female mean shapes differ significantly within each strain (*p*<0.0001 for all 9 tests).

The differences between the three species averages in full shape space are summarized in Figure 3B. Besides the eye-to-face ratio, *D. melanogaster* also differs from the other two species by more ventrally displaced bristles (see black arrowheads in Figure 3B). A permutation test confirms that mean shapes differ significantly between strains (*p*<0.0002 for all 36 pair-wise tests except for Kib32/YVF which is not significant).

Finally our analysis of different head proportions also revealed that *D. mauritiana* eyes are not uniformly larger than the eyes of the other two species but that the dorsal portion of the *D. mauritiana* eye in particular is larger compared to the eyes of *D. simulans* (not shown) and *D. melanogaster* (Figure 3C). Manual area measurements also show that the dorsal region of the eye is significantly larger in *D. mauritiana* than *D. simulans* (pairwise Wilcoxon rank test: W = 74, p = 0.00042) or *D. melanogaster* (pairwise Wilcoxon rank test: W = 55, p<0.0001) (Figure 3D). A Kruskal-Wallis rank test applied to the whole dataset also shows significant differences between mean ranks of d-v ratios (*X^2^* = 18.138, df = 2, *p* = 0.00012).

### Dorsal enlargement of *D. mauritiana* eyes is associated with an increase in the expression of *rhodopsin3*

Given the differences we have found in eye size and shape, we next analyzed the expression of all seven *rhodopsin* genes at the mRNA level using quantitative real-time PCR.

**Figure 4:**
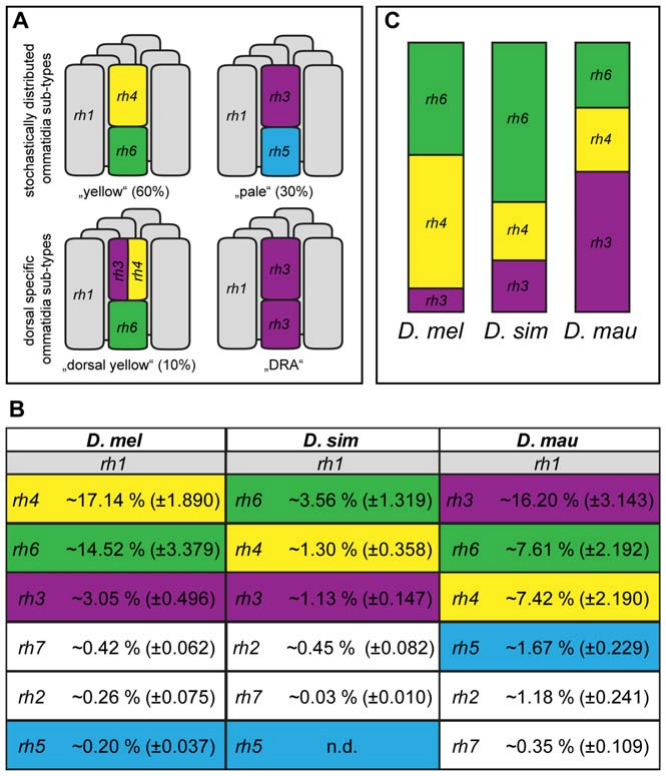
*rhodopsin* expression among *Drosophila* species. **A.** Schematic representation of four different ommatidia subtypes in *D. melanogaster* [73]. The six outer photoreceptors (R 1–6) express *rhodopsin 1* (grey, *rh1*) and the inner photoreceptors (R7 on top of R8) express subtype-specific combinations of *rhodopsin* genes. DRA = dorsal rim area ommatidia. **B.** Schematic representation of the quantitative real-time PCR results for *rh3*, *rh4* and *rh6*. The respective portion of each bar represents the relative expression as shown in C. Therefore, the sum of percentages in each column is not comparable between species. **C.** Comprehensive summary of quantitative real-time PCR results. Mean percentages based on 10 to 12 replicate measurements are given relative to *rh1* expression. Standard deviation is given in brackets. Note that we were not able to detect expression of *D. simulans rh5* (n.d.). *rh2* is specifically expressed in the dorsal ocelli [85] and the function of *rh7* is unknown.

*rh1* is expressed in all outer photoreceptor cells (R1–6) (Figure 4A), and as expected exhibits the highest expression level in all three species (not shown). For *D. melanogaster*, it is expected that *rh4* and *rh6* are both highly expressed because they are co-expressed in “yellow” and dorsal “yellow” ommatidia types, which contribute to approximately 70% of the whole eye (Figure 4A) [26], [27], [30]. We found similar relative expression levels for those two *rhodopsins* in *D. melanogaster* and *D. mauritiana* (Figure 4B,C; *rh4/rh6* = 1.18 and 0.96 for *D. melanogaster* and *D. mauritiana*, respectively). Interestingly, the co-expression of these two *rhodopsins* seems to be uncoupled in *D. simulans* because we found relatively higher expression levels of *rh6* than *rh4* (Figure 4B,C; *rh4/rh6* = 0.37 for *D. simulans*). In *D. melanogaster, rh3*, which is predominantly expressed in pale and in dorsal-specific ommatidia types (dorsal “yellow” types and dorsal rim ommatidia), should be more abundant than *rh5* whose expression is restricted to 30% “pale” type ommatidia [26], [27], [29], [30], [33], [34]. This expectation is met in *D. melanogaster* since *rh3* expression exceeds the levels of *rh5* (Figure 4B, C) and, moreover, both mRNAs are less abundant than *rh1*, *rh4* and *rh6* (Figure 4B,C).

Strikingly *rh3* expression is greatly enhanced in *D. mauritiana* compared to the other two species (Figure 4B,C). High expression of *rh3* in *D. mauritiana* could be explained by the higher expression of *rh5* in this species (Figure 4B,C) indicating an enrichment of “pale” ommatidia types (co-expressing *rh3* and *rh5*) or that there an enrichment of dorsal-specific ommatidia types in this species.

## Discussion

### Inter-specific and intra-specific variation in eye size in *Drosophila*

We have found extensive variation in eye size within and among three closely related *Drosophila* species. *D. mauritiana* generally tends to have larger eyes than its sister species *D. melanogaster* and *D. simulans* as a consequence of increases in either ommatidia number as previously reported by Hammerle and Ferrus (2003) [35] and/or ommatidia size. We did detect variation in both of these traits among *D. mauritiana* strains suggesting that there is intra-specific variation in these traits in this species. For example, the finding that *D. mauritiana* is capable of producing larger eyes than *D. melanogaster* as a consequence of larger ommatidia in the case of TAM16, but more ommatidia in MAV1 shows that eye size differences can be the result of changes in various different traits within the eye. Indeed, we found a white-eyed mutant strain of this species with eyes more similar in size to those of *D. simulans* strains. Therefore, the large eyes of *D. mauritiana* may overlap with the range of eye sizes observed in *D. simulans*, for which our broader survey suggests extensive intra-specific variation in eye size (Figure 1B, S2).

The large variation in eye size among *D. melanogaster* strains mainly owes to differences in ommatidia number. The most recently collected African strain of *D. melanogaster* (Zi372) clearly has larger eyes than either OreR and M36, which is consistent with evidence showing that the longer flies live in captivity the smaller their eyes and body size become, probably due to relaxed selection on vision [36]. This may also be a possible explanation for the small eyes of the *D. mauritiana w^−^* strain. However, it cannot be excluded that the loss of function of the *white* gene might interfere with normal eye development because in recent years several new functions of this ABC-transporter protein have been found including an involvement in cyclic GMP transport [62] courtship behaviour [63], [64] and in learning an memory [65], [66].

The formation of larger eyes based on larger ommatidia observed in some strains of *D. mauritiana* suggests that their vision might be specialized to achieve higher sensitivity at the expense of acuity [9]. A similar trend has been reported for nocturnal bee and crepuscular butterfly species, which form larger eyes due to larger facets compared to diurnal species [13], [67]. In addition, the dorsal enlargement of *D. mauritiana* eyes may indicate that *D. mauritiana* has a locally specialized zone in the dorsal-frontal portion of the eye field, and although this remains to be tested, our measurements of *rhodopsin* expression suggests a dorsal specialization based on specific ommatidia types (see below). Intriguingly, such specializations in vision are often correlated with adaptation to a given sensory niche [13], [67]–[70], but little is known regarding the relationship between vision and environment in *Drosophila*.

### Evolution and development of head capsule proportions

Differences in eye size between strains and species are part of more global differences in the whole head: flies that have larger eyes have narrower faces and vice versa, both in terms of absolute size and relative size. This is most strikingly illustrated by PC 1 (Figure 3A), which clearly separates all strains by their species including *D. mauritiana white-*, which does not have particularly large eyes.

A negative correlation between eye width and face width has previously been reported in *D. melanogaster* and between the two cactophilic sibling species *Drosophila buzzatii* and *Drosophila koepferae* [16], [18]. Since the eyes and face cuticle both develop from the eye antennal disc [17], it has been suggested that changes in the relative proportions of these tissues represent a developmental trade-off mediated by differences in the morphogens *wingless* (*wg*) and *decapentaplegic* (*dpp*) that are expressed in the anterior and posterior region of the eye-antennal disc [15], [71]. These changes in the *Drosophila* head may be analogous to those in horned beetles, where exaggerated development of cephalic horns results in the reduction of the eyes [72]. However, it remains possible that the differences in the eye and face size of *Drosophila* are evolving independently and have a different genetic basis. For example, the reduction of the face in some blowflies is not associated with an increase in overall eye size [14], and in stalk-eyed flies the distance between the eyes appears to be subject to sexual selection [73]. Intriguingly, we also found that in all surveyed *Drosophila* strains females have relatively larger eyes, containing more and larger ommatidia, and wider faces than males, as previously reported for *D. melanogaster* [74], again suggesting a decoupling of developmental programs for these two tissues.

Therefore, the observed variation in eye and face size could be due to coupled developmental changes or independent evolution of these tissues. Can these two developmental scenarios be distinguished using our dataset? It has been proposed that scaling relationships allow inferences about the developmental mechanism underlying a given trait [60], [61], [75]. For example, differences in the slope of linear relationships between body size and the trait of interest could be the result of differences in starting conditions before organ growth commences. Differences in the y-intercept of linear relationships with constant slopes might hint at different proliferation rates during the growth phase of the given organ [60]. We find that the enlarged eyes of *D. mauritiana* are positively allometric, whereas the eyes are negatively allometric in *D. melanogaster* and *D. simulans* (Fig. 1C). The differences in the slope could suggest that during the early subdivision of the eye-antennal disc more cells are allocated to the retinal field than to the presumptive face tissue in *D. mauritiana* as compared to *D. melanogaster* or *D. simulans* [15]. Thus, upon proliferation, the eye-antennal disc of *D. mauritiana* would produce more cells with a retinal fate relative to face cuticle in the adult. However, this is likely to be overly simplistic since the eye size differences we have observed are a consequence of changes in both ommatidia size and ommatidia number along both the a-p and d-v axes, suggesting that multiple developmental mechanisms are involved.

### Evolution of *rhodopsin* expression

In addition to changes in eye size and shape, we also found differences in the relative expression of *rhodopsin* genes between *D. mauritiana*, *D. melanogaster* and *D. simulans*.

We found enhanced *rh3* and *rh5* expression in *D. mauritiana* compared to *D. melanogaster*, suggesting an enrichment of “pale” ommatidia in *D. mauritiana* eyes. However, the formation of more “pale” ommatidia alone does not account for the high *rh3* expression in *D. mauritiana* eyes. This suggests that *D. mauritiana* might contain more dorsal-specific ommatidia types and is consistent with our observation that the eyes of *D. mauritiana* are enlarged dorsally compared to those of the other species.

These possibly coordinated differences in the eyes of *D. mauritiana* have potentially interesting implications for the vision of this species. An enrichment of dorsal “yellow” ommatidia that co-express *rh3* and *rh4* in the R7 cell would result in increased sensitivity to UV light used for navigation [33]. More monochromic DRA ommatidia on the other hand would provide higher light sensitivity and polarized light detection [76]–[79]. However, our finding that *D. mauritiana* tends to have larger overall eye size suggests a need for increased light sensitivity perhaps due to a more crepuscular life style.

Although the stochastic distribution of “yellow” and “pale” type ommatidia is conserved in other dipterans like blowflies and houseflies [22], [80], the fact that dorsal “yellow” ommatidia are able to co-express different rhodopsins in R7 implies there is a level of plasticity. It has recently been shown that “yellow” R8 PRs in the dorsal portion of the eye retain the capability to co-express Rh5 and Rh6 when reared in complete darkness [81]. This suggests that the visual system is flexible enough to allow the rapid adaptation to changing environments by new visual properties. Given the co-expression of *rh4* in R7 and *rh6* in R8 in *D. melanogaster* “yellow” and dorsal “yellow” ommatidia subtypes [26], [27], [30], [33], the observed uncoupled activity of those two *rhodopsin* genes in *D. simulans* may suggest that the ommatidia sub-type composition has also changed in this species. In fact, atypical coupling of Rh3 and Rh6 occurs in ∼6% of all ommatidia in wild type compound eyes without a restriction to the dorsal portion of the eye field [27], [33], [82]–[84].

### Conclusions

We have found extensive intra- and inter-specific variation in eye morphology among three *Drosophila* species, which are part of more large-scale differences in head tissue proportions, and in some cases are associated with changes in *rhodopsin* expression, and therefore, relative populations of ommatidia subtypes. Mapping the genetic basis of these differences represents an excellent opportunity to better understand the evolutionary changes in the development and function of this complex organ, and contribute to explaining the great diversity in compound eyes among insects.

## Supporting Information

Figure S1 **Overview of wing landmarks.** *D. melanogaster* wing showing the 15 landmarks, which were used to calculate different parameters of wing size.
(TIFF)

Figure S2 **Eye size variation in different strains of** ***D. simulans*** **and** ***D. mauritiana*** **.** Variation in eye width in several strains of *D. simulans* and *D. mauritiana*. Eye width is reported as residuals of a regression of eye width and wing length to account for variation in body size. Each strain is represented by five males and five females.
(TIF)

Figure S3 **Face width variation in three** ***Drosophila*** **species.** Variation in face width (FW in Figure 1A) in *D. melanogaster* M36, *D. simulans* YVF and *D. mauritiana* TAM16. Face width is given as residuals of a regression of face width and tibia length to account for variation in body size. Each strain is represented by 40 to 65 females.
(TIF)

Table S1 Pair-wise comparisons of eye size variation defined as multiple comparisons of means (Tukey comparisons).
(DOC)

Table S2 Summary of mean number of ommatidia (ommatidia), ommatidia size (µm^2^), and number of antero-posterior (A-P) and dorso-ventral (D-V) ommatidia rows. Standard deviation is given in parentheses. Note that ommatidia size was estimated by dividing the eye area by the number of ommatidia.
(DOC)

Table S3 Sample size and average relative eye size (ratio of eye size to face size) for each sex, strain, and species, respectively.
(DOC)
