# Correction: Evolution of Eye Morphology and Rhodopsin Expression in the *Drosophila melanogaster* Species Subgroup

**DOI:** 10.1371/annotation/a72ae6bf-afe8-4244-b175-e303f02686f8

**Published:** 2012-07-10

**Authors:** Nico Posnien, Corinna Hopfen, Maarten Hilbrant, Margarita Ramos-Womack, Sophie Murat, Anna Schönauer, Samantha L. Herbert, Maria D. S. Nunes, Saad Arif, Casper J. Breuker, Christian Schlötterer, Philipp Mitteroecker, Alistair P. McGregor

There was a error in the equal contributions designation and in the corresponding address of the second author in the online version of the article. Nico Posnien and Corinna Hopfen contributed equally to this work. The listed current address is for Corinna Hopfen. This information in the PDF version of the paper is correct. 

