# Supplementary figures and images for: Evolution of Eye Morphology and Rhodopsin Expression in the Drosophila melanogaster Species Subgroup
Source: PLoS One. 2012 May 25;7(5):e37346. doi: 10.1371/journal.pone.0037346 (PMC3360684; doi:10.1371/journal.pone.0037346)

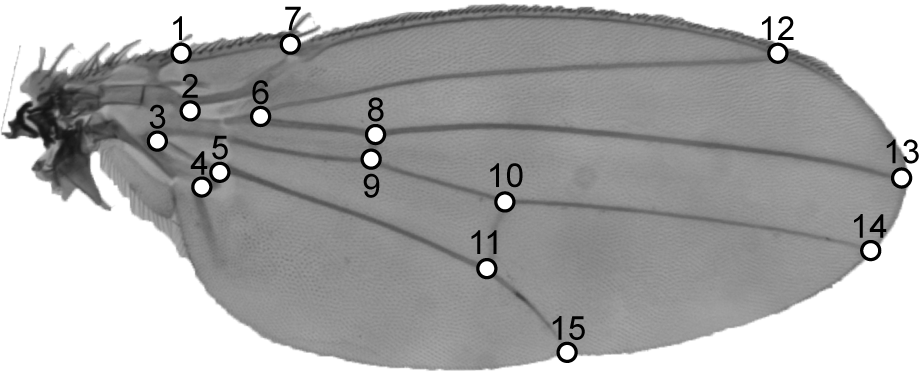

Supplement: Figure S1 — Overview of wing landmarks. D. melanogaster wing showing the 15 landmarks, which were used to calculate different parameters of wing size. (TIFF) [file pone.0037346.s001.tif]

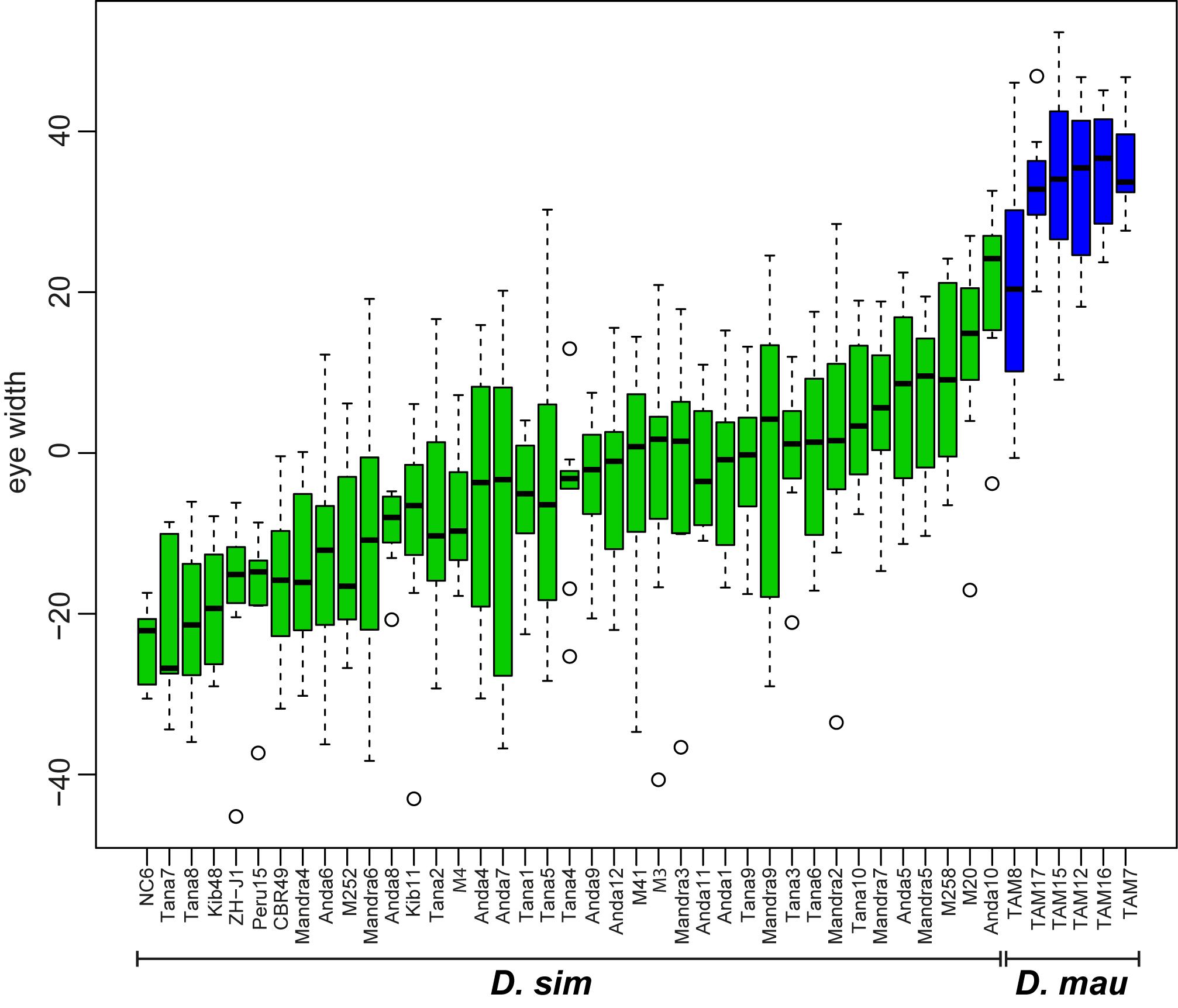

Supplement: Figure S2 — Eye size variation in different strains of D. simulans and D. mauritiana . Variation in eye width in several strains of D. simulans and D. mauritiana. Eye width is reported as residuals of a regression of eye width and wing length to account for variation in body size. Each strain is represented by five males and five females. (TIF) [file pone.0037346.s002.tif]

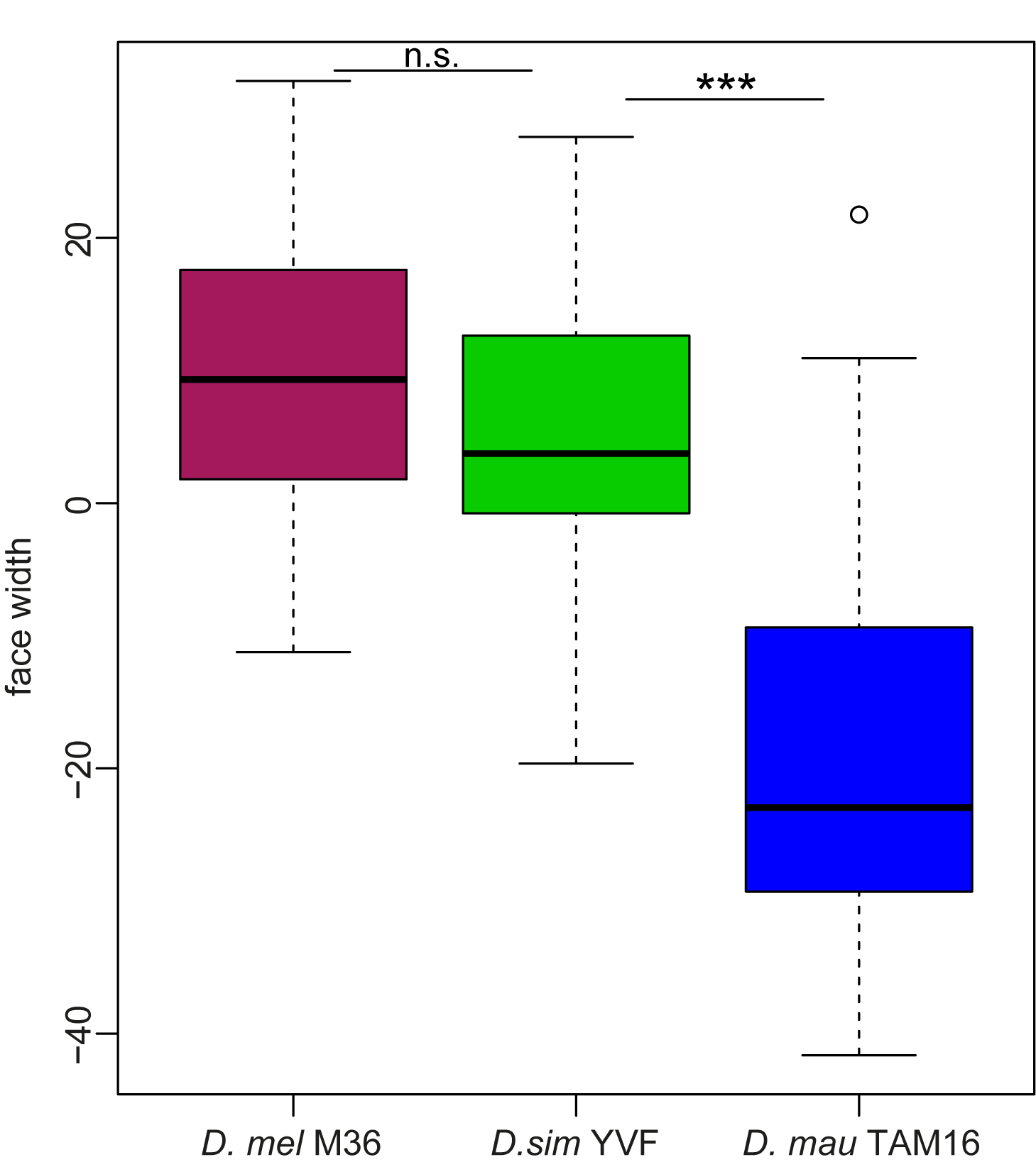

Supplement: Figure S3 — Face width variation in three Drosophila species. Variation in face width (FW in Figure 1A) in D. melanogaster M36, D. simulans YVF and D. mauritiana TAM16. Face width is given as residuals of a regression of face width and tibia length to account for variation in body size. Each strain is represented by 40 to 65 females. (TIF) [file pone.0037346.s003.tif]
