# Supplementary material for: Evolution of Eye Morphology and Rhodopsin Expression in the Drosophila melanogaster Species Subgroup
Source: PLoS One. 2012 May 25;7(5):e37346. doi: 10.1371/journal.pone.0037346 (PMC3360684; doi:10.1371/journal.pone.0037346)
Supplement: Table S1 — Pair-wise comparisons of eye size variation defined as multiple comparisons of means (Tukey comparisons). (DOC) [file pone.0037346.s004.doc]

**Table S1.** Pairwise comparisons of eye area variation defined as multiple comparisons of means (ANOVA: F(17,597) = 168.37; p < 0.0001).

Legend: F = femal, M = male

| **Level 1** | **Level 2** | **p-value** | **Significance** |
| --- | --- | --- | --- |
| D. mau_white-_M | D. mau_white-_F | <0.01 | *** |
| D. mau_MAV1_F | D. mau_white-_F | <0.01 | *** |
| D. mau_MAV1_F | D. mau_white-_M | <0.01 | *** |
| D. mau_MAV1_M | D. mau_white-_F | 0,6577 |  |
| D. mau_MAV1_M | D. mau_white-_M | <0.01 | *** |
| D. mau_MAV1_M | D. mau_MAV1_F | <0.01 | *** |
| D. mau_TAM16_F | D. mau_white-_F | <0.01 | *** |
| D. mau_TAM16_F | D. mau_white-_M | <0.01 | *** |
| D. mau_TAM16_F | D. mau_MAV1_F | <0.01 | *** |
| D. mau_TAM16_F | D. mau_MAV1_M | <0.01 | *** |
| D. mau_TAM16_M | D. mau_white-_F | <0.01 | *** |
| D. mau_TAM16_M | D. mau_white-_M | <0.01 | *** |
| D. mau_TAM16_M | D. mau_MAV1_F | 0,7464 |  |
| D. mau_TAM16_M | D. mau_MAV1_M | <0.01 | *** |
| D. mau_TAM16_M | D. mau_TAM16_F | <0.01 | *** |
| D. mel_M36_F | D. mau_white-_F | 1 |  |
| D. mel_M36_F | D. mau_white-_M | <0.01 | *** |
| D. mel_M36_F | D. mau_MAV1_F | <0.01 | *** |
| D. mel_M36_F | D. mau_MAV1_M | 0,3743 |  |
| D. mel_M36_F | D. mau_TAM16_F | <0.01 | *** |
| D. mel_M36_F | D. mau_TAM16_M | <0.01 | *** |
| D. mel_M36_M | D. mau_white-_F | <0.01 | *** |
| D. mel_M36_M | D. mau_white-_M | <0.01 | *** |
| D. mel_M36_M | D. mau_MAV1_F | <0.01 | *** |
| D. mel_M36_M | D. mau_MAV1_M | <0.01 | *** |
| D. mel_M36_M | D. mau_TAM16_F | <0.01 | *** |
| D. mel_M36_M | D. mau_TAM16_M | <0.01 | *** |
| D. mel_M36_M | D. mel_M36_F | <0.01 | *** |
| D. mel_OreR_F | D. mau_white-_F | 0,8446 |  |
| D. mel_OreR_F | D. mau_white-_M | 0,0767 | . |
| D. mel_OreR_F | D. mau_MAV1_F | <0.01 | *** |
| D. mel_OreR_F | D. mau_MAV1_M | <0.01 | ** |
| D. mel_OreR_F | D. mau_TAM16_F | <0.01 | *** |
| D. mel_OreR_F | D. mau_TAM16_M | <0.01 | *** |
| D. mel_OreR_F | D. mel_M36_F | 0,9151 |  |
| D. mel_OreR_F | D. mel_M36_M | <0.01 | *** |
| D. mel_OreR_M | D. mau_white-_F | <0.01 | *** |
| D. mel_OreR_M | D. mau_white-_M | <0.01 | *** |
| D. mel_OreR_M | D. mau_MAV1_F | <0.01 | *** |
| D. mel_OreR_M | D. mau_MAV1_M | <0.01 | *** |
| D. mel_OreR_M | D. mau_TAM16_F | <0.01 | *** |
| D. mel_OreR_M | D. mau_TAM16_M | <0.01 | *** |
| D. mel_OreR_M | D. mel_M36_F | <0.01 | *** |
| D. mel_OreR_M | D. mel_M36_M | <0.01 | *** |
| D. mel_OreR_M | D. mel_OreR_F | <0.01 | *** |
| D. mel_Zi372_F | D. mau_white-_F | <0.01 | *** |
| D. mel_Zi372_F | D. mau_white-_M | <0.01 | *** |
| D. mel_Zi372_F | D. mau_MAV1_F | 0,0502 | . |
| D. mel_Zi372_F | D. mau_MAV1_M | <0.01 | *** |
| D. mel_Zi372_F | D. mau_TAM16_F | <0.01 | *** |
| D. mel_Zi372_F | D. mau_TAM16_M | 1 |  |
| D. mel_Zi372_F | D. mel_M36_F | <0.01 | *** |
| D. mel_Zi372_F | D. mel_M36_M | <0.01 | *** |
| D. mel_Zi372_F | D. mel_OreR_F | <0.01 | *** |
| D. mel_Zi372_F | D. mel_OreR_M | <0.01 | *** |
| D. mel_Zi372_M | D. mau_white-_F | <0.01 | *** |
| D. mel_Zi372_M | D. mau_white-_M | 1 |  |
| D. mel_Zi372_M | D. mau_MAV1_F | <0.01 | *** |
| D. mel_Zi372_M | D. mau_MAV1_M | <0.01 | *** |
| D. mel_Zi372_M | D. mau_TAM16_F | <0.01 | *** |
| D. mel_Zi372_M | D. mau_TAM16_M | <0.01 | *** |
| D. mel_Zi372_M | D. mel_M36_F | <0.01 | *** |
| D. mel_Zi372_M | D. mel_M36_M | <0.01 | *** |
| D. mel_Zi372_M | D. mel_OreR_F | 0,2143 |  |
| D. mel_Zi372_M | D. mel_OreR_M | <0.01 | *** |
| D. mel_Zi372_M | D. mel_Zi372_F | <0.01 | *** |
| D. sim_Kib32_F | D. mau_white-_F | <0.01 | *** |
| D. sim_Kib32_F | D. mau_white-_M | <0.01 | *** |
| D. sim_Kib32_F | D. mau_MAV1_F | <0.01 | *** |
| D. sim_Kib32_F | D. mau_MAV1_M | 0,3905 |  |
| D. sim_Kib32_F | D. mau_TAM16_F | <0.01 | *** |
| D. sim_Kib32_F | D. mau_TAM16_M | 0,2452 |  |
| D. sim_Kib32_F | D. mel_M36_F | <0.01 | *** |
| D. sim_Kib32_F | D. mel_M36_M | <0.01 | *** |
| D. sim_Kib32_F | D. mel_OreR_F | <0.01 | *** |
| D. sim_Kib32_F | D. mel_OreR_M | <0.01 | *** |
| D. sim_Kib32_F | D. mel_Zi372_F | 0,4706 |  |
| D. sim_Kib32_F | D. mel_Zi372_M | <0.01 | *** |
| D. sim_Kib32_M | D. mau_white-_F | <0.01 | *** |
| D. sim_Kib32_M | D. mau_white-_M | 1 |  |
| D. sim_Kib32_M | D. mau_MAV1_F | <0.01 | *** |
| D. sim_Kib32_M | D. mau_MAV1_M | <0.01 | *** |
| D. sim_Kib32_M | D. mau_TAM16_F | <0.01 | *** |
| D. sim_Kib32_M | D. mau_TAM16_M | <0.01 | *** |
| D. sim_Kib32_M | D. mel_M36_F | <0.01 | *** |
| D. sim_Kib32_M | D. mel_M36_M | <0.01 | *** |
| D. sim_Kib32_M | D. mel_OreR_F | 0,2928 |  |
| D. sim_Kib32_M | D. mel_OreR_M | <0.01 | *** |
| D. sim_Kib32_M | D. mel_Zi372_F | <0.01 | *** |
| D. sim_Kib32_M | D. mel_Zi372_M | 1 |  |
| D. sim_Kib32_M | D. sim_Kib32_F | <0.01 | *** |
| D. sim_W501_F | D. mau_white-_F | <0.01 | *** |
| D. sim_W501_F | D. mau_white-_M | <0.01 | *** |
| D. sim_W501_F | D. mau_MAV1_F | <0.01 | *** |
| D. sim_W501_F | D. mau_MAV1_M | 0,0141 | * |
| D. sim_W501_F | D. mau_TAM16_F | <0.01 | *** |
| D. sim_W501_F | D. mau_TAM16_M | 0,8565 |  |
| D. sim_W501_F | D. mel_M36_F | <0.01 | *** |
| D. sim_W501_F | D. mel_M36_M | <0.01 | *** |
| D. sim_W501_F | D. mel_OreR_F | <0.01 | *** |
| D. sim_W501_F | D. mel_OreR_M | <0.01 | *** |
| D. sim_W501_F | D. mel_Zi372_F | 0,9891 |  |
| D. sim_W501_F | D. mel_Zi372_M | <0.01 | *** |
| D. sim_W501_F | D. sim_Kib32_F | 0,9998 |  |
| D. sim_W501_F | D. sim_Kib32_M | <0.01 | *** |
| D. sim_W501_M | D. mau_white-_F | 0,1269 |  |
| D. sim_W501_M | D. mau_white-_M | 0,6602 |  |
| D. sim_W501_M | D. mau_MAV1_F | <0.01 | *** |
| D. sim_W501_M | D. mau_MAV1_M | <0.01 | *** |
| D. sim_W501_M | D. mau_TAM16_F | <0.01 | *** |
| D. sim_W501_M | D. mau_TAM16_M | <0.01 | *** |
| D. sim_W501_M | D. mel_M36_F | 0,1636 |  |
| D. sim_W501_M | D. mel_M36_M | <0.01 | *** |
| D. sim_W501_M | D. mel_OreR_F | 0,9998 |  |
| D. sim_W501_M | D. mel_OreR_M | <0.01 | *** |
| D. sim_W501_M | D. mel_Zi372_F | <0.01 | *** |
| D. sim_W501_M | D. mel_Zi372_M | 0,8985 |  |
| D. sim_W501_M | D. sim_Kib32_F | <0.01 | *** |
| D. sim_W501_M | D. sim_Kib32_M | 0,9336 |  |
| D. sim_W501_M | D. sim_W501_F | <0.01 | *** |
| D. sim_YVF_F | D. mau_white-_F | <0.01 | *** |
| D. sim_YVF_F | D. mau_white-_M | <0.01 | *** |
| D. sim_YVF_F | D. mau_MAV1_F | 0,1691 |  |
| D. sim_YVF_F | D. mau_MAV1_M | <0.01 | *** |
| D. sim_YVF_F | D. mau_TAM16_F | <0.01 | *** |
| D. sim_YVF_F | D. mau_TAM16_M | 1 |  |
| D. sim_YVF_F | D. mel_M36_F | <0.01 | *** |
| D. sim_YVF_F | D. mel_M36_M | <0.01 | *** |
| D. sim_YVF_F | D. mel_OreR_F | <0.01 | *** |
| D. sim_YVF_F | D. mel_OreR_M | <0.01 | *** |
| D. sim_YVF_F | D. mel_Zi372_F | 1 |  |
| D. sim_YVF_F | D. mel_Zi372_M | <0.01 | *** |
| D. sim_YVF_F | D. sim_Kib32_F | 0,3311 |  |
| D. sim_YVF_F | D. sim_Kib32_M | <0.01 | *** |
| D. sim_YVF_F | D. sim_W501_F | 0,9531 |  |
| D. sim_YVF_F | D. sim_W501_M | <0.01 | *** |
| D. sim_YVF_M | D. mau_white-_F | 0,5658 |  |
| D. sim_YVF_M | D. mau_white-_M | 0,4038 |  |
| D. sim_YVF_M | D. mau_MAV1_F | <0.01 | *** |
| D. sim_YVF_M | D. mau_MAV1_M | <0.01 | *** |
| D. sim_YVF_M | D. mau_TAM16_F | <0.01 | *** |
| D. sim_YVF_M | D. mau_TAM16_M | <0.01 | *** |
| D. sim_YVF_M | D. mel_M36_F | 0,6726 |  |
| D. sim_YVF_M | D. mel_M36_M | <0.01 | *** |
| D. sim_YVF_M | D. mel_OreR_F | 1 |  |
| D. sim_YVF_M | D. mel_OreR_M | <0.01 | *** |
| D. sim_YVF_M | D. mel_Zi372_F | <0.01 | *** |
| D. sim_YVF_M | D. mel_Zi372_M | 0,6819 |  |
| D. sim_YVF_M | D. sim_Kib32_F | <0.01 | *** |
| D. sim_YVF_M | D. sim_Kib32_M | 0,7527 |  |
| D. sim_YVF_M | D. sim_W501_F | <0.01 | *** |
| D. sim_YVF_M | D. sim_W501_M | 1 |  |
| D. sim_YVF_M | D. sim_YVF_F | <0.01 | *** |
