# Supplementary material for: Evolution of Eye Morphology and Rhodopsin Expression in the Drosophila melanogaster Species Subgroup
Source: PLoS One. 2012 May 25;7(5):e37346. doi: 10.1371/journal.pone.0037346 (PMC3360684; doi:10.1371/journal.pone.0037346)
Supplement: Table S2 — Summary of mean number of ommatidia (ommatidia), ommatidia size (µm2), and number of antero-posterior (A-P) and dorso-ventral (D-V) ommatidia rows. Standard deviation is given in parentheses. Note that ommatidia size was estimated by dividing the eye area by the number of ommatidia. (DOC) [file pone.0037346.s005.doc]

**Table S2**. Summary of mean number of ommatidia (ommatidia), ommatidia size (µm^2^), number of antero-posterior ommatidia rows (A-P rows) and number dorso-ventral ommatidia rows (D-V rows). Standard deviation is given in parentheses. Note that ommatidia size was estimated by dividing the eye area by the number of ommatidia.

| **Species** | ***D. melanogaster*** | | | | ***D. simulans*** | | ***D. mauritiana*** | |
| --- | --- | --- | --- | --- | --- | --- | --- | --- |
| **Strain** | **M36** | | **Zi375** | | **YVF** | | **TAM16** | |
| **Sex** | females | males | females | males | females | males | females | males |
| **Ommatidia** | 785 (4.7) | 728.8 (23.09) | 905.8 (40.37) | 814.8 (24.6) | 975.5 (45.52) | 898.2 (30.02) | 957 (22.89) | 890.5 (15.25) |
| **Ommatidia size** | 174.7 (7.86) | 161.4 (7.12) | 168.6 (8.96) | 159.1 (11.8) | 150.9 (7.03) | 147.5 (4.61) | 185.5 (2.8) | 170.8 (5.03) |
| **A-P rows** | 27.4 (0.55) | 26.5 (0.55) | 29 (0.81) | 27.2 (0.45) | 30.3 (0.82) | 30.2 (1.3) | 30.6 (0.51) | 29.5 (0.52) |
| **D-V rows** | 36 (0) | 35.2 (0.75) | 40 (1.41) | 38.4 (0.55) | 41.8 (0.75) | 40.2 (1.3) | 39.8 (0.75) | 38.6 (0.81) |
