# Supplementary material for: Evolution of Eye Morphology and Rhodopsin Expression in the Drosophila melanogaster Species Subgroup
Source: PLoS One. 2012 May 25;7(5):e37346. doi: 10.1371/journal.pone.0037346 (PMC3360684; doi:10.1371/journal.pone.0037346)
Supplement: Table S3 — Sample size and average relative eye size (ratio of eye size to face size) for each sex, strain, and species, respectively. (DOC) [file pone.0037346.s006.doc]

**Table S3**. Sample size and average relative eye size (ratio of eye size to face size) for each sex, strain, and species, respectively.

| **Strain** | **Sample size** | **Size ratio sex** | **Size ratio strain** | **Size ratio species** |
| --- | --- | --- | --- | --- |
| M36 females | 29 | 0.347 | 0.351 | 0.360 |
| M36 males | 18 | 0.356 |  |  |
| OreR femals | 33 | 0.334 | 0.341 |  |
| OreR males | 35 | 0.349 |  |  |
| Zi372 females | 36 | 0.379 | 0.390 |  |
| Zi372 males | 36 | 0.401 |  |  |
| YVF females | 9 | 0.440 | 0.446 | 0.420 |
| YVF males | 7 | 0.453 |  |  |
| w501 females | 36 | 0.393 | 0.402 |  |
| w501 males | 36 | 0.412 |  |  |
| Kib32 females | 32 | 0.404 | 0.413 |  |
| Kib32 males | 32 | 0.422 |  |  |
| TAM16 females | 32 | 0.474 | 0.489 | 0.473 |
| TAM16 males | 23 | 0.505 |  |  |
| *white-* females | 35 | 0.431 | 0.445 |  |
| *white-* males | 35 | 0.459 |  |  |
| MAV1 females | 35 | 0.474 | 0.484 |  |
| MAV1 males | 32 | 0.495 |  |  |
